# Voriconazole Pharmacokinetics Are Not Altered in Critically Ill Patients with Acute-on-Chronic Liver Failure and Continuous Renal Replacement Therapy: An Observational Study

**DOI:** 10.3390/microorganisms9102087

**Published:** 2021-10-03

**Authors:** Jörn Grensemann, Christoph Pfaffendorf, Sebastian G. Wicha, Christina König, Kevin Roedl, Dominik Jarczak, Stefanie Iwersen-Bergmann, Carolin Manthey, Stefan Kluge, Valentin Fuhrmann

**Affiliations:** 1 Department of Intensive Care Medicine, University Medical Center Hamburg-Eppendorf, Martinistraße 52, 20246 Hamburg, Germany; ch.koenig@uke.de (C.K.); k.roedl@uke.de (K.R.); d.jarczak@uke.de (D.J.); s.kluge@uke.de (S.K.); vfuhrmann@outlook.de (V.F.); 2 Department of Clinical Pharmacy, Institute of Pharmacy, University of Hamburg, Bundesstrasse 45, 20146 Hamburg, Germany; christoph.pfaffendorf@uni-hamburg.de (C.P.); sebastian.wicha@uni-hamburg.de (S.G.W.); 3 Hospital Pharmacy, University Medical Center Hamburg-Eppendorf, Martinistraße 52, 20246 Hamburg, Germany; 4 Department of Legal Medicine, University Medical Center Hamburg-Eppendorf, Butenfeld 34, 22529 Hamburg, Germany; s.iwersen-bergmann@uke.de; 5 First Department of Internal Medicine and Gastroenterology, University Medical Center Hamburg-Eppendorf, Martinistraße 52, 20246 Hamburg, Germany; c.manthey@uke.de; 6 Department of Gastroenterology, Protestant-Hospital Duisburg-Nord, Fahrner Straße 133, 47169 Duisburg, Germany

**Keywords:** antifungal therapy, target attainment, intensive care, volume of distribution, Monte-Carlo simulation, population pharmacokinetics, probability of target attainment

## Abstract

Infection and sepsis are a main cause of acute-on-chronic liver failure (ACLF). Besides bacteria, molds play a role. Voriconazole (VRC) is recommended but its pharmacokinetics (PK) may be altered by ACLF. Because ACLF patients often suffer from concomitant acute renal failure, we studied the PK of VRC in patients receiving continuous renal replacement therapy (RRT) with ACLF and compared it to PK of VRC in critically ill patients with RRT without concomitant liver failure (NLF). In this prospective cohort study, patients received weight-based VRC. Pre- and post-dialysis membrane, and dialysate samples obtained at different time points were analyzed by high-performance liquid chromatography. An integrated dialysis pharmacometric model was used to model the available PK data. The recommended, 50% lower, and 50% higher doses were analyzed by Monte-Carlo simulation (MCS) for day 1 and at steady-state with a target trough concentration (TC) of 0.5–3mg/L. Fifteen patients were included in this study. Of these, 6 patients suffered from ACLF. A two-compartment model with linear clearance described VRC PK. No difference for central (V1) or peripheral (V2) volumes of distribution or clearance could be demonstrated between the groups. V1 was 80.6L (95% confidence interval: 62.6–104) and V2 106L (65–166) with a body clearance of 4.7L/h (2.87–7.81) and RRT clearance of 1.46L/h (1.29–1.64). MCS showed TC below/within/above target of 10/74/16% on day 1 and 9/39/52% at steady-state for the recommended dose. A 50% lower dose resulted in 26/72/1% (day 1) and 17/64/19% at steady-state and 7/57/37% and 7/27/67% for a 50% higher dose. VRC pharmacokinetics are not significantly influenced by ACLF in critically ill patients who receive RRT. Maintenance dose should be adjusted in both groups. Due to the high interindividual variability, therapeutic drug monitoring seems inevitable.

## 1. Introduction

Within the group of critically ill patients, patients with acute-on-chronic liver failure (ACLF) are particularly susceptible to infections associated with a high mortality [1,2,3,4,5,6]. Besides bacterial pathogens, molds and other fungi may play a role [7,8,9]. Voriconazole is recommended as first line treatment against *Aspergillus* spp. and is also active against *Scedosporium*, *Fusarium* spp. and yeasts [10,11]. Voriconazole is extensively metabolized by cytochrome enzymes with 2% renal excretion of the unmetabolized drug [12]. Its volume of distribution (V) has been estimated at approximately 200 L [13,14], but V may increase as a result of capillary leak syndrome and ascites [15], necessitating higher doses. Contrarily, elimination may be decreased due to liver failure, thus requiring lower doses.

Due to these complex pharmacokinetics and further dependency on cytochrome isotypes, therapeutic drug monitoring (TDM) has been recommended to adjust therapy [11], but sufficient initial dosing strategies could prevent patients from receiving inadequate serum values before TDM results are available and the dose is adjusted.

The most severely ill ACLF patients often suffer from acute kidney injury as well [3] and require renal replacement therapy [16,17]. Therefore, we studied the impact of ACLF on PK of voriconazole in critically ill patients requiring continuous renal replacement therapy (RRT) in comparison to critically ill patients receiving RRT without ACLF.

## 2. Materials and Methods

### 2.1. Ethics

The study was approved by the Ethics Committee of the Hamburg Chamber of Physicians, Germany (Reference: PV5415). Consent was obtained from the patients’ closest relatives or legal surrogates.

### 2.2. Study Design

Patients eligible for this open label observational prospective cohort study were receiving voriconazole for clinical indication and required RRT. Patients < 18 years or with an extracorporeal circuit other than the RRT were excluded. Patients were grouped according to liver function as follows: patients with ACLF and patients without ACLF (“no liver failure”, NLF).

### 2.3. Liver Cirrhosis and ACLF

ACLF was defined according to the definition of the Chronic Liver Failure (CLIF) Consortium [3]. Presence of liver cirrhosis was diagnosed based on a combination of clinical characteristic (e.g., ascites, caput medusae, spider angiomata, etc.), laboratory and radiological findings (typical morphological changes of the liver, signs of portal hypertension, etc. in ultrasonography or computed tomography scanning), or via histology, if available [18].

### 2.4. Medication

All patients received voriconazole (Dr. Friedrich Eberth Arzneimittel GmbH, Ursensollen, Germany) adapted to body weight or prior serum levels. An initial dose of 6 mg/kg for two doses followed by 4 mg/kg was targeted. Voriconazole was diluted in 50 mL isotonic saline solution and given over 30 min by syringe pump via a central venous line (short-term infusion).

### 2.5. Sampling and Storage

We obtained dialysis circuit pre- and postfilter blood samples as well as ultrafiltrate samples at the following time points: T0 as the baseline before the first monitored infusion, 1 h (T1), 2 h (T2), 4 h (T4), 6 h (T6), 8 h (T8), and 12 h after the start of infusion (T12). T12 was obtained before the next infusion of voriconazole as trough concentration. Furthermore, we obtained values after 24 h (immediately before start of infusion, T24) and 25 h (30 min after end of infusion, T25) and after 48 h and 49 h (T48 and T49). All samples were centrifuged immediately, and supernatants were stored at −20 °C until assayed.

### 2.6. Assay

Quantification of voriconazole in serum was performed by high performance liquid chromatography (HPLC) using a commercially available, fully validated, and IVD-CE-labeled kit (Itraconazole, Posaconazole and Voriconazole in serum/plasma—HPLC. Order Number 27037; ChromSystems Instruments & Chemicals GmbH, Graefelfing, Germany). This assay used the reagents, controls mobile phase and HPLC column provided by the ChromSystems HPLC Kit for TDM of voriconazole. Chromatographic separation was performed on a Thermo Scientifc Dionex UltiMate 3000 chromatography system consisting of an autosampler, quaternary pump, a fluorescence detector, and a diode array detector (Thermo Fisher Scientific, Dreieich, Germany). Isocratic flow rate was set at 1.4 mL/min. Detection was performed using a fluorescence detector with excitation wavelength set at 261 nm and emission wavelength set at 366 nm, and also a Diode array detector.

### 2.7. Renal Replacement Therapy

RRT was performed as continuous veno-venous hemodialysis (CVVHD) or as a postdilution continuous veno-venous hemodiafiltration (CVVH). Both methods were performed with Multifiltrate^®^ dialysis machines using an Ultraflux^®^ AV1000S hollow-fiber hemofilter (Fresenius Medical Care, Bad Homburg, Germany) with a membrane surface area of 1.8 m^2^. Dialyzers and lines were steam sterilized. For CVVHD, a regional citrate-calcium anticoagulation was used; and the targeted dialysate dose was 30 mL/kg/h of actual body weight. CVVH was chosen in cases of severe acidosis due to the technically higher possible blood flow. No filter change occurred during the study period.

### 2.8. Patient Characteristics

Additional data were obtained from the patients’ electronic records (Integrated Care Manager ICM, version 9.1, Drägerwerk, Lübeck, Germany, and Soarian Clinicals 4.01 SP08, Cerner Health Services, Idstein, Germany).

The Acute Physiology And Chronic Health Evaluation II (APACHE II) score [19] and the Sequential Organ Failure Assessment (SOFA) score [20] were recorded on the first day of examination as measures of disease severity. ACLF patients were further characterized by the Model of End-Stage Liver Disease (MELD) score, the Chronic Liver Failure Consortium (CLIF)-SOFA score, and the CLIF-Lactate-Score [18].

### 2.9. Statistics

Microsoft Excel 2016 (Microsoft Corp., Redmond, WA, USA) was used for data management. The SPSS statistical software package (version 25, IBM Inc., Armonk, NY, USA) was used for descriptive statistical analysis.

### 2.10. Pharmacometric Data Analysis

The integrated dialysis pharmacometric (IDP) model was used to model the available pharmacokinetic (PK) data [21]. The IDP model allows to integrate available parameters of the modality of renal replacement therapy (RRT) as well as the pre-filter, post-filter, and effluent voriconazole concentration to discriminate between the body clearance and the RRT clearance. Furthermore, pre-, post-filter, and effluent concentration can be simultaneously considered, which allows a quantitative estimation of potential adsorption processes of voriconazole to the hemofilter.

One- and two-compartment models with linear and non-linear (Michaelis–Menten) elimination were evaluated to describe the pre-filter plasma concentration time courses. Interindividual variability (IIV) was investigated on all structural parameters. For the residual variability, additive and proportional error models as well as a combination of these were assessed.

The RRT clearance was estimated as follows: The pre- post-filter concentration-based RRT clearance was:

(1) $$Q_{\mathrm{blood}\;\mathrm{adj}.}=Q_{\mathrm{blood}}\times \left(1-\mathrm{Hct}+\mathrm{Hct}\;\times \mathrm{RBCtP}\right)$$

(2) $${\mathrm{CL}}_{\mathrm{pre}-\mathrm{postfilter}}={\;Q}_{\mathrm{blood}\;\mathrm{adj}.}\times \frac{C_{\mathrm{pl}\left(\mathrm{pre}\right)}-{\;C}_{\mathrm{pl}\left(\mathrm{post}\right)}}{C_{\mathrm{pl}\left(\mathrm{pre}\right)}}$$

(3) $$C_{\mathrm{pl}\left(\mathrm{post}\right)\;\mathrm{corr}.}={\;C}_{\mathrm{pl}\left(\mathrm{post}\right)\mathrm{meas}.}\times \frac{Q_{\mathrm{blood}\;\mathrm{adj}.\;}-{\;Q}_{\mathrm{FRR}}}{Q_{\mathrm{blood}\;\mathrm{adj}.}}$$

with the adjusted blood flow rate (Q_blood adj._) being calculated using the blood flow (Q_blood_), the hematocrit (Hct) and the red blood cell-to-plasma-ratio (RBCtP), with C_pl(pre)_ representing the pre-filter and C_pl(post)_ the post-filter plasma concentration.

The effluent concentration-based RRT clearance was:

(4) $${\mathrm{CL}}_{\mathrm{pre}-\mathrm{effluent}}={\;Q}_{\mathrm{effl}.\;}\times \frac{C_{\mathrm{effl}.}}{C_{\mathrm{pl}\left(\mathrm{pre}\right)}}$$

(5) $$Q_{\mathrm{effl}.}={\;Q}_{\mathrm{dial}}+{\;Q}_{\mathrm{RF}\;\mathrm{pre}}+{\;Q}_{\mathrm{FRR}}$$

with Q_effl_ representing the total effluent flow rate, Q_dial_ the dialysate flow rate, Q_RF pre_ the pre-filter replacement fluid flow rate, Q_FRR_ the fluid removal rate, and C_effl._ the concentration of drug in the effluent and C_pl(pre)_ the pre-filter plasma concentration.

The IDP model was used with an adsorption compartment to quantify adsorption processes, parameterized from potential time-dependent differences between the post-filter and effluent-based RRT clearance. Thus, the IDP model allowed us to subdivide the RRT clearance into the dialysis clearance CL_dial_ and the clearance caused by adsorption on the hemofilter CL_ads_. A detailed description of the IDP model is provided elsewhere [21].

Different candidate models were developed including no adsorption, reversible vs. irreversible adsorption, as well as capacity-limited adsorption vs. no adsorption limit. The developed models were compared numerically by using the objection function value and/or the Akaike information criterion (AIC) as well as graphically using residual plots, visual predictive checks, as well as overlay plots of individually predicted vs. observed PK measurements to assess the model performance in the individual patients. The parameter uncertainty was determined by the log-likelihood-profiling-based sampling importance resampling (LLP-SIR) technique for accurate confidence interval determination in small datasets [22].

Covariate model building was performed via stepwise covariate model building (SCM). The significance level was 0.05 for the forward inclusion and 0.01 for the backward elimination steps.

The individual PK parameters (empirical Bayesian estimates for each individual patient) for each group were analyzed. Potential differences in the mean of the PK and RRT parameters between the ACLF and the NLF groups for each parameter were tested using either the t-test or the Wilcoxon test.

### 2.11. Simulations

The best-performing model was used for Monte Carlo simulations using the typical PK parameters and the estimated variability components of the model. A total of 9 different scenarios was simulated, i.e., three dose levels and three RRT modalities, as follows: Three different dose levels were simulated:

▪ The recommended standard dosing regimen which included an initial dose of 6 mg/kg/12 h of voriconazole on the first day and a maintenance dose of 4 mg/kg/12 h on each subsequent day,
▪ a regimen with 50% higher doses compared to the standard dosing regimen (loading dose: 9 mg/kg/12 h, maintenance dose: 6 mg/kg/12 h), and
▪ a regimen with 50% lower doses compared to the standard dosing regimen (loading dose: 3 mg/kg/12 h, maintenance dose: 2 mg/kg/12 h).

Each dose was administered as a bolus infusion. Each scenario was simulated with (i) a continuous RRT using the dialysis parameters derived from the study population, (ii) a continuous RRT including a filter change to estimate the potential impact of drug adsorption on the PK profile and (iii) a scenario without RRT to estimate the effect of RRT on the PK profile. Trough concentration and an area under curve (AUC) to minimal inhibitory concentration (MIC) ratio were calculated and used to evaluate PK/PD target attainment because AUC/MIC ratios have been shown to be predictive of voriconazole treatment efficacy [23]. Trough concentrations below 0.5 mg/L were assumed to be associated with a loss of efficacy and trough concentrations above 3 mg/L and 4 mg/L with an increase of hepatotoxicity and neurotoxicity, respectively. Thus, the target trough plasma concentration was defined to be between 0.5 and 3 mg/L [24]. In vitro experiments with Aspergillus fumigatus have shown that an AUC_24h_ to MIC ratio of >32 is an effective exposure level for the treatment with voriconazole [25]. Hence, probability of target attainment (PTA) was calculated using the target trough concentration as well as the AUC_24h_ to MIC ratio at MIC values ranging from 0.125 to 32 mg/L in two-fold dilution steps.

## 3. Results

A total of fifteen critically ill patients were included in this study with six patients suffering from ACLF and renal failure and nine patients from renal failure, only. ACLF patients had a mean MELD score of 32 (30–34), a CLIF-SOFA of 19 (17–20) and a CLIF-Lactate-Score of 57 (54–60). An overview on patients’ characteristics is given in Table 1.

Four ACLF patients were admitted for gastrointestinal hemorrhage and two for septic shock. Microbiological sampling revealed *Aspergillus fumigatus* in four patients, *Clavispora* spp. in one patient, and no fungi in one patient. In the NLF group, four patients were treated for pneumonia, two patients for anastomotic insufficiency after esophageal resection, and one patient each for hemorrhagic shock from retroperitoneal hematoma after kidney biopsy, urosepsis, and hypovolemic shock from exsiccation. *Aspergillus* spp. were identified in three cases, galactomannan was positive in one case, and calculated antifungal therapy was commenced in five cases.

All patients suffering from ACLF (100%) and five (56%) patients in the NLF group died during the intensive care stay (*p* = 0.06).

### 3.1. Pharmacometric Data Analysis

Voriconazole plasma PK was better described by a two-compartment model than a one-compartment model. Linear elimination was chosen as non-linear elimination showed implausible parameter estimates. The estimation of the RBCtP ratio to calculate the Q_blood adj._ improved the model performance. When RBCtP was set to zero, the model systematically overpredicted the pre-filter and underpredicted the post-filter voriconazole concentrations. A significant amount of voriconazole was estimated to reside in the red blood cells as a typical RBCtP ratio of 2.13 was estimated. Adding an adsorption fraction as a component of RRT clearance improved the model fit. A maximum adsorbed voriconazole amount of 29–160 mg was estimated, indicating that a small fraction of the RRT clearance might be mediated by adsorption of voriconazole to the hemofilter. IIV was supported for CL_body_, V1, Q, V2, CL_RRT_, and the fraction of the RRT clearance mediated by adsorption (F_ADS_). Except for the interindividual variability of CL_RRT_ (17.7% CV), the IIV for CL_body_ (95.3% CV), V1 (45.3% CV), Q (76.9% CV), V2 (74.7% CV), F_ADS_ (50.1% CV) was estimated to be very high. Additionally, CL_body_ varied substantially across dosing occasions with an IOV CL_body_ of 83.8% CV. The final estimates for all parameters are presented in the Table 2.

Potential covariate effects were evaluated using the SCM procedure, which was performed for ACLF, age, and sex on V1, V2, Q, and the body clearance. No significant effects of the covariates on any of the parameters was found. Therefore, no covariate was not included in the final model.

Due to the study aims, the impact of ACLF was explored in more detail: Although not statistically significant, there was a trend for a lower body clearance in the ACLF group (2.95 vs. 4.83 L/h). Conversely, the RRT clearance tended to be higher in the ACLF group (1.34 vs. 1.01 L/h). The difference in the RRT clearance was driven by a slightly higher saturation coefficient (0.25 vs. 0.2) rather than different blood or dialysis flow rates between the patient groups. However, none of these trends in the parameters between ACLF and NLF patients were significant. The results of the analysis are shown in Table 3. The prediction-corrected visual predictive checks indicate a very good predictive performance for all pre-, post-filter, and effluent voriconazole concentrations.

### 3.2. Simulations

**Figure 1:**
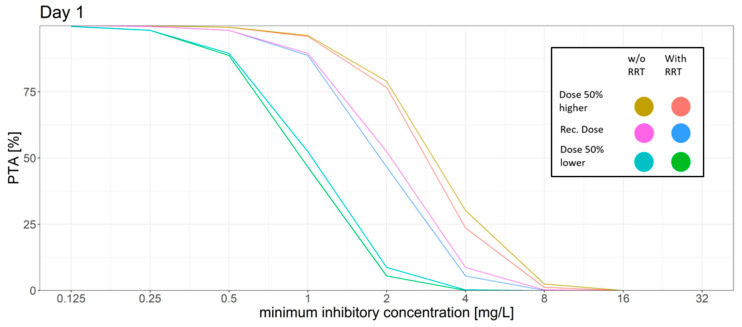
Different dosing scenarios and their probability of target attainment (PTA) for different MICs on day 1. An AUC to MIC ratio of >32 was set as the target. Recommended dose: initial dose: 6 mg/kg/12 h; maintenance dose: 4 mg/kg/12 h, 50% higher dose: initial dose: 9 mg/kg/12 h; maintenance dose: 6 mg/kg/12 h, 50% lower dose: initial dose: 3 mg/kg/12 h, maintenance dose: 2 mg/kg/12 h.

**Figure 2:**
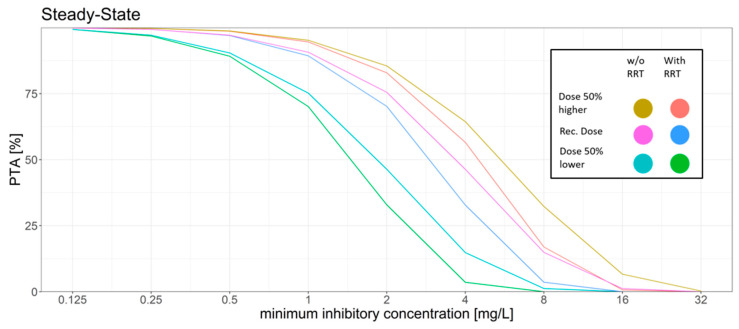
Different dosing scenarios and their probability of target attainment (PTA) for different MICs at steady-state. An AUC to MIC ratio of >32 was set as the target. Recommended dose: initial dose: 6 mg/kg/12 h; maintenance dose: 4 mg/kg/12 h, 50% higher dose: initial dose: 9 mg/kg/12 h; maintenance dose: 6 mg/kg/12 h, 50% lower dose: initial dose: 3 mg/kg/12 h, maintenance dose: 2 mg/kg/12 h.

The summaries of the Monte Carlo simulations with and without RRT for the PTA for the AUC_24h_/MIC target at different MIC values on day 1 and day 6 (steady state) are shown in Figure 1 and Figure 2, and the trough concentration target attainment for efficacy and toxicity is presented in Table 4 and Table 5 (for day 1 and 6, respectively).

For the standard dosing regimen, the Monte Carlo simulations revealed that for the AUC_24h_/MIC target a high PTA was attained for MIC values ≤ 1 mg/L (PTA > 89%). MIC values above 1 mg/L led to a rapid decrease in the PTA. The trough concentration target for efficacy was attained in most of the patients with only 10% on day 1 and 9% on day 6 of the patients not attaining the target. However, trough concentration levels had a high probability of surpassing the breakpoints for increased hepatotoxicity and neurotoxicity. 52% of the simulated patients had a trough concentration above 3 mg/L on day 6.

The dosing regimen with a 50% higher dose led to slight increase of the PTA for the AUC_24h_/MIC target with a PTA of >94% at an MIC of 1 mg/L and a slight decrease of the probability of patients having a trough concentration below the efficacy threshold (7%). However, the probability of trough concentration above 3 mg/L increased from 52% to 67% on day 6 as compared to the standard dosing regimen.

The dosing regimen with a 50% reduced dose showed a decrease of the probability of toxic trough concentration at only 19% vs. 52% on day 6 compared to the standard dosing regimen. This coincided with a higher fraction of patients with trough concentration below the efficacy threshold (17%, day 6) and the AUC_24h_/MIC target for efficacy was only reached with a probability of ca. 90% for MIC values ≤ 0.5 mg/L.

The introduction of a regular filter change had little to no effect on target attainment in this scenario (data not shown).

Simulation of RRT vs. no RRT led to a slight decrease in the PTA while also decreasing the probability of reaching toxic trough concentrations.

## 4. Discussion

In this study, we assessed the impact of ACLF on PK and PTA of voriconazole in critically ill patients undergoing RRT. Although voriconazole is mainly hepatically metabolized, we could not show a difference between the ACLF and NLF group.

Our findings are contradictory to our hypothesis that liver failure would reduce the predominant metabolism of voriconazole by the cytochrome P450 enzymes (CYP), mainly CYP2C19 as well as CYP3A4 and CYP2C9 [26]. While, e.g., CYP2C19 has been shown to be of significant influence on PK [27], no effect of CYP genotype could be shown in another evaluation [28]. According to the label of voriconazole, a 50% dose reduction for liver cirrhosis Child-Pugh A and B is recommended while no data are available on Child-Pugh C patients. This dose reduction in liver cirrhosis relies on case reports [14,29] and retrospective observations [27,30,31].

However, critically ill patients were not included in these studies. Often, these patients also suffer from renal failure and require RRT [3,16]. Under normal circumstances, the renal elimination of non-metabolized voriconazole is negligible (approximately 2%) [23]. Elimination of voriconazole by continuous RRT has been shown to be approximately 1 L/h [14] and is thus higher than the renal elimination in healthy volunteers [32]. In our study, elimination was even higher with approximately 1.5 L/h, which we attribute to the larger hemofilter membrane area as compared to the type used in the previous study. Compared to the body clearance of voriconazole, clearance by RRT accounts for approximately one-fourth of the total clearance in this patient population, which can be regarded as a clinically relevant proportion. On the other hand, our simulations only revealed a minor difference in PTA for the scenarios with and without RRT.

Interestingly, our IDP model indicates that a small amount of voriconazole may be adsorbed to the hemofilter membrane. So far, only a sequestration of voriconazole into ECMO-membranes has been shown [33,34], but conflicting data exist [35]. The adsorption to hemofilter membranes should be elucidated further in the future, as an additional dose for each filter change might help to provide more consistent plasma levels.

Hypoalbuminemia was present in both groups, but according to the voriconazole label, hepatic impairment does not alter plasma protein binding. Of the plasma protein binding, 25% of voriconazole are bound to albumin, 5% to α_1_-acid glycoprotein, and the remaining 70% are unknown [36].

The voriconazole solution used in this study contains hydroxypropyl-beta-cyclodextrin (HPBCD) as solubilizer. Its physicochemical properties are similar to the widely used solubilizer sulfobutylether-beta-cyclodextrin (SEBCD) that may cumulate in renal failure due to its nearly exclusive renal elimination [37] and the manufacturer recommends giving voriconazole as tablets. However, in critically ill patients, oral application is not feasible and it has been shown that cyclodextrines are effectively removed by RRT [38,39,40]. Therefore, we chose not to measure HPBCD concentrations.

In an in vitro model, voriconazole has been shown to require AUC/MIC ratio of 55 or 32 for the suppression of galactomannan depending on the type of methodology used [25]. This leads to break-points for susceptibility of 0.5 mg/L for the Clinical Laboratory Standards Institute (CLSI) and 1.0 mg/L for the European Committee of Antimicrobial Susceptibility Testing (EUCAST) methodology. These target values are concordant to clinical data. A meta-analysis could establish a dose response relationship between voriconazole concentrations and clinical success [41]. The authors conclude that targeting a concentration between 1.0 and 6.0 mg/L optimizes success and limits toxicity. In another meta-analysis, a target trough concentration between 0.5 and 3.0 mg/L has been associated with the lowest mortality, lowest hepatotoxicity, and lowest neurotoxicity [24]. For these analyses minimal inhibitory concentrations of the targeted molds have not been considered, but the EUCAST epidemiological cut-off value (ECOFF) as the highest typical minimal inhibitory concentration equals the breakpoint and therefore, only few resistant strains should be expected.

Concerning our modelling of PTA for different doses at the beginning of therapy, the recommended dose yielded few patients above and below the targeted concentration. Neither increasing nor decreasing the initial dose optimized the PTA. At a steady state, the unadjusted dose as per labelling of voriconazole resulted in more than half of patients with toxic trough concentrations. Halving the maintenance dose as suggested in the labelling for liver cirrhosis grade Child-Pugh A and B still resulted in more than one-third of patients in the toxic range. Of note, according to our data this dose adjustment seems reasonable for our patient population irrespective of concomitant liver failure. The addition of RRT had a small but noticeable influence on PTA and should be considered in clinical practice. That the trough concentrations achieved are unpredictable is attributable to the high interindividual variability. Therefore, TDM seems inevitable for the safety and efficacy of voriconazole in critically ill patients [42].

Our study has certain limitations. First, the number of patients was small and heterogeneous, and this necessarily limits the precision of the PK parameters and the variability components of the model. However, this is a common number of patients for pharmacokinetic studies and the first study assessing PK data in ACLF. We did not obtain polymorphism status on cytochrome enzymes which may influence PK of voriconazole. However, polymorphism diagnostics are usually not available before TDM and therefore of only little value in clinical practice. The calculated amount of adsorbed drug relies on accurate calculation of pre-postfilter vs. effluent-based clearance and we did not obtain measurements from cumulated effluent to verify dialysis flow rates. Concerning the necessary target concentrations, the AUC/MIC target has only been shown in in vitro experiments, but the resulting breakpoints have been verified in clinical studies. We did not measure the free fraction of voriconazole that might be increased by hypoalbuminemia, resulting in possible toxicity [43]. However, only 25% of voriconazole is bound to albumin [36].

## 5. Conclusions

Voriconazole PK in critically ill patients undergoing continuous RRT is not significantly influenced by ACLF. Contrarily to other patient groups, one quarter of the total clearance occurs via RRT. Furthermore, a small amount of voriconazole is adsorbed to the hemofilter membrane. According to our modelling analysis, the recommended initial dose should not be adjusted, however, the maintenance dose may be decreased irrespective of concomitant liver failure. Due to the high interindividual variability with a significant number of patients above toxic or below effective trough concentrations, TDM seems inevitable when prescribing voriconazole in this critically ill patient population and should be commenced as soon as feasible.

## Figures and Tables

**Table 1:** Patients’ characteristics.

Parameter	ACLF	NLF	*p*
**Number of patients**	*n* = 6	*n* = 9	
**Age [years]**	58 (49–67)	70 (53–73)	0.17
**Gender**	males: 4 females: 2	males: 9 females: 0	0.06
**Weight [kg]**	73 (64–103)	85 (76–95)	0.33
**Height [cm]**	175 (164–181)	180 (173–182)	0.39
**APACHE II**	30 (23–40)	25 (21–39)	0.69
**SOFA**	19 (15–23)	13 (10–18)	0.11
**Albumin**	16.1 (11.1–19.5)	12.0 (10.5–15.6)	0.35
**PT [%]**	33 (23–82)	86 (61–104)	0.05
**Bilirubin [mg/dL]**	10.3 (3.5–14.2)	1.1 (0.6–1.4)	0.001
**Antithrombin [%]**	24 (21–86)	99 (50–106)	0.07

ACLF: Acute-on-chronic liver failure due to liver cirrhosis; NLF: patients without liver failure; APACHE II: Acute Physiology And Chronic Health Evaluation; SOFA: sequential organ failure assessment score; PT: prothrombin time; data are given as median and quartiles.

**Table 2:** Parameter estimates of the pharmacometric model for voriconazole in patients undergoing RRT.

Parameter	Estimate	CI_95%_
**CL_body_ [L/h]**	4.70	2.87–7.81
**V1 [L]**	80.6	62.6–104
**Q [L/h]**	62.1	36.3–110
**V2 [L]**	106	65.0–166
**CLRRT [L/h]**	1.46	1.29–1.64
**F_ADS_**	0.23	0.13–0.33
**ADS_MAX_ [mg]**	69.2	29.3–159
**RBCtP**	2.13	1.43–30
**IIV CL_body_ [%CV]**	95.3	67.4–135
**IIV V1 [%CV]**	45.2	32.6–59.7
**IIV Q [%CV]**	76.9	30.8–151
**IIV V2 [%CV]**	74.7	44.2–120.8
**IIV CLRRT [%CV]**	17.7	12.1–25.8
**IIV FADS [%CV]**	50.1	24.3–92.9
**IOV CL_body_ [%CV]**	83.8	75.5–95.1
**RUV pre, prop. [%CV]**	29.3	27.2–31.3
**RUV post, prop [%CV]**	35.9	34.0–38.5
**RUV dia, prop [%CV]**	42.9	40.1–46.3

CL_body_: body clearance; V1: central volume of distribution; Q: intercompartmental clearance, V2: peripheral volume of distribution; CL_RRT_: clearance by renal replacement therapy; F_ADS_: fraction of RRT clearance mediated by adsorption; ADS_MAX_: maximum amount of drug adsorbed on filter membrane; RBCtP: red blood cell to plasma ratio; IIV: interindividual variability; IOV: interoccasion variability; RUV: residual variability; pre: pre-filter plasma concentration; post: post-filter concentration, dia: dialysate concentration; CV: coefficient of variation; CI_95%_: 95% confidence interval determined by log-likelihood profiling-based sampling importance resampling.

**Table 3:** Parameter estimates of the pharmacometric model for patients with and without ACLF. Normal distributed parameters are reported as mean [sd] and non-normal as median [min, max].

Parameter	ACLF	NLF	*p*-Value
**Number of patients**	*n* = 6	*n* = 9	
**CL_body_ [L/h]**	2.95 [1.24, 12.14]	4.83 [2.54, 31.96]	0.181
**V1 [L]**	88.88 [27.81]	85.14 [42.2]	0.85
**Q [L/h]**	77.63 [61.89, 82.34]	68.55 [75.79]	0.69
**V2 [L]**	122.59 [36.94]	120.17 [155.62]	0.97
**CL_RRT_ [L/h]**	1.34 [0.94, 1.82]	1.01 [0.78, 1.44]	0.14
**S_eff_**	0.25 [0.06]	0.2 [0.09]	0.31

CL_body_: body clearance; V1: central volume of distribution; Q: intercompartmental clearance, V2: peripheral volume of distribution; CL_RRT_: clearance by renal replacement therapy; S_eff_: saturation coefficient of effluent.

**Table 4:** Probability of target attainment for the trough concentration-based breakpoints for efficacy, increased risk of hepatotoxicity, and neurotoxicity for day 1 of the dosing regimens.

Scenario	Cmin < 0.5 mg/L (Loss of Efficacy)	Cmin 0.5–3 mg/L (Target Range)	Cmin > 3 mg/L (Increased Incidence of Hepatotoxicity)	Cmin > 4 mg/L (Increased Incidence of Neurotoxicity)
**Initial dose: 6 mg/kg/12 h (2×)** **Maintenance dose:** **4 mg/kg/12 h** **(Recommended dose)**	10%	74%	16%	7%
**Initial dose: 9 mg/kg/12 h (2×)** **Maintenance dose:** **6 mg/kg/12 h**	7%	57%	37%	21%
**Initial dose: 3 mg/kg/12 h (2×)** **Maintenance dose:** **2 mg/kg/12 h**	26%	72%	1%	0%
**Initial dose: 6 mg/kg/12 h (2×)** **Maintenance dose:** **4 mg/kg/12 h** **(Recommended dose)** **w/o RRT**	9%	68%	22%	11%
**Initial dose: 9 mg/kg/12 h (2×)** **Maintenance dose:** **6 mg/kg/12 h** **w/o RRT**	6%	51%	43%	28%
**Initial dose: 3 mg/kg/12 h (2×)** **Maintenance dose:** **2 mg/kg/12 h** **w/o RRT**	23%	74%	3%	1%

Cmin: trough concentration; RRT: renal replacement therapy.

**Table 5:** Probability of target attainment for the trough concentration-based breakpoints for efficacy, increased risk of hepatotoxicity, and neurotoxicity for day 6 of the dosing regimens.

Scenario	Cmin < 0.5 mg/L (Loss of Efficacy)	Cmin 0.5–3 mg/L (Target Range)	Cmin > 3 mg/L (Increased Incidence of Hepatotoxicity)	Cmin > 4 mg/L (Increased Incidence of Neurotoxicity)
**Initial dose: 6 mg/kg/12 h (2×)** **Maintenance dose:** **4 mg/kg/12 h** **(Recommended dose)**	9%	39%	52%	39%
**Initial dose: 9 mg/kg/12 h (2×)** **Maintenance dose:** **6 mg/kg/12 h**	7%	27%	67%	57%
**Initial dose: 3 mg/kg/12 h (2×)** **Maintenance dose:** **2 mg/kg/12 h**	17%	64%	19%	8%
**Initial dose: 6 mg/kg/12 h (2×)** **Maintenance dose:** **4 mg/kg/12 h** **(Recommended dose)** **w/o RRT**	8%	31%	61%	51%
**Initial dose: 9 mg/kg/12 h (2×)** **Maintenance dose:** **6 mg/kg/12 h** **w/o RRT**	6%	22%	73%	65%
**Initial dose: 3 mg/kg/12 h (2×)** **Maintenance dose:** **2 mg/kg/12 h** **w/o RRT**	15%	51%	35%	23%

Cmin: trough concentration; RRT: renal replacement therapy.

## Data Availability

Data are available from the authors upon reasonable request.
